# Long-term survival after neoadjuvant therapy for triple-negative breast cancer under different treatment regimens: a systematic review and network meta-analysis

**DOI:** 10.1186/s12885-024-12222-9

**Published:** 2024-04-09

**Authors:** Zhilin Liu, Jinming Li, Fuxing Zhao, Dengfeng Ren, Zitao Li, Yongzhi Chen, Shifen Huang, Zhen Liu, Yi Zhao, Miaozhou Wang, Huihui Li, ZhengBo Xu, Guoshuang Shen, Jiuda Zhao

**Affiliations:** 1 Breast Disease Diagnosis and Treatment Center, Affiliated Hospital of Qinghai University, Affiliated Cancer Hospital of Qinghai University, People’s Republic of China, Qinghai Provincial Clinical Research Center for Cancer, Qinghai Provincial Institute of Cancer Research, Xining, China; 2 Department of Breast Medical Oncology, Shandong Cancer Hospital and Institute, Shandong First Medical University and Shandong Academy of Medical Sciences, Jinan, China; 3 Qinghai University, Xining, China

**Keywords:** Triple negative breast cancer, Neoadjuvant therapy, Long-term survival, Network meta-analysis

## Abstract

**Background:**

Triple-negative breast cancer (TNBC) is a life-threatening subtype of breast cancer with limited treatment options. Therefore, this network meta-analysis (NMA) aimed to evaluate and compare the effect of various neoadjuvant chemotherapy (NCT) options on the long-term survival of patients with TNBC.

**Methods:**

PubMed, Embase, Medline, Cochrane Library, Web of Science, and major international conference databases were systematically searched for randomized controlled trials (RCTs) on the efficacy of various NCT options in patients with TNBC. Searches were performed from January 2000 to June 2023. Study heterogeneity was assessed using the I^2^ statistic. Hazard ratios (HRs) and 95% confidence intervals (CIs) were used to evaluate disease-free survival (DFS) and overall survival (OS). Odds ratios (ORs) and 95% CIs were used to evaluate the pathologic complete response (pCR). The primary outcome was DFS.

**Results:**

We conducted an NMA of 21 RCTs involving 8873 patients with TNBC. Our study defined the combination of anthracyclines and taxanes as the preferred treatment option. On this basis, the addition of any of the following new drugs is considered a new treatment option: bevacizumab (B), platinum (P), poly-ADP-ribose polymerase inhibitors (PARPi), and immune checkpoint inhibitor (ICI). Based on the surface under the cumulative ranking curve (SUCRA) values, the top three SUCRA area values of DFS were taxanes, anthracycline, and cyclophosphamide (TAC; 89.23%); CT (84.53%); and B (81.06%). The top three SUCRA area values of OS were CT (83.70%), TAC (62.02%), and B-containing regimens (60.06%). The top three SUCRA area values of pCR were B + P-containing regimens (82.7%), ICI + P-containing regimens (80.2%), and ICI-containing regimens (61.8%).

**Conclusions:**

This NMA showed that standard chemotherapy is a good choice with respect to long-term survival. Moreover, B associated with P-containing regimens is likely to be the optimal treatment option for neoadjuvant TNBC in terms of pCR.

## Introduction

The latest global cancer burden data released by the World Health Organization International Agency for Research on Cancer in 2020 indicated that the number of new breast cancer cases reached 2.26 million worldwide, exceeding the total number (2.2 million) of lung cancer cases [1]. Breast cancer has replaced lung cancer to become the world’s most prevalent cancer [2]. It poses a great threat to the physical and mental health of patients worldwide. Breast cancer treatment is a very long and complex process, and the cost is also very high, and even some patients give up treatment because they cannot afford the treatment cost, and further worsen the condition. Triple-negative breast cancer (TNBC) is a subtype of breast cancer characterized by the lack of receptor-estrogen and progesterone expression and amplification of human epidermal growth factor receptor 2 [3, 4]. Clinically, TNBC is one of the most aggressive subtypes of breast cancer, accounting for approximately 15%–20% of all breast cancers [5]. Endocrine therapy with hormone receptor and targeted therapy to block human epidermal growth factor receptor 2 (HER-2) have proven ineffective for patients with TNBC [6]. The clinical course of TNBC is aggressive, with a high probability of visceral and brain metastases, and its prognosis is the worst among the breast cancer subtypes [7, 8]. The BRCA 1/2 gene is particularly strongly associated with triple-negative breast cancer. In the Chinese population, the BRCA 1/2 mutation rate is less than 1% in the general population and about 3% in all breast cancer patients, and up to 17.3% in triple-negative breast cancer. From another perspective, approximately 60%-80% of breast cancer patients carrying the BRCA 1 mutation are triple-negative breast cancer, while approximately 25% of breast cancer patients carrying the BRCA 2 mutation have triple-negative breast cancer [9, 10].

Anthracyclines, cyclophosphamides, and taxanes are the preferred neoadjuvant chemotherapy (NCT) for TNBC [11, 12]. NCT can reduce the micrometastasis, shrink the tumor, reduce the stage, and increase the chance of breast preservation treatment, which improve the radical cure and breast preservation rate and obtain the drug sensitivity information [13, 14]. Studies confirm that achieving pathological complete response (pCR) after a neoadjuvant treatment with TNBC has a good predictive value for long-term survival benefits [15]. Currently, platinum (P) and poly-ADP-ribose polymerase inhibitors (PARPi) play important antitumor roles in NCT for TNBC, and their efficacy is significant in young patients, especially with BRCA gene mutations. As a DNA cross-linking agent, P cross-connects with the DNA after entering the tumor cells, which interferes with DNA replication of the tumor cells, leading to double-strand DNA breaks of the tumor cells, and then killing the tumor cells. Several single-arm or randomized controlled clinical studies including GeparSixto, CALGB40603, BrighTNess, NeoCART have confirmed the efficacy and safety of P-containing chemotherapy regimens for the treatment of TNBC [16–19].

Immune checkpoint inhibitor (ICI) therapy is directed against the interaction between the programmed death protein 1 (PD-1) and programmed death ligand 1 (PD-L1) [20, 21]. PD-1 is a co-inhibitory molecule expressed by activated T cells when antigen-presenting cells or tumor cells are combined with PD-L1, which further lead to inhibiting the T-cell activation and suppressing the body’s antitumor immune response. Moreover, the view of PD-1/PD-L1 ICI can improve the suppressed antitumor immune response to relieve the body’s immune response inhibition state, further realizing the antitumor effects [22, 23]. ICI may enhance the endogenous anticancer immunity after increasing the release of tumor-specific antigens through chemotherapy. Most current studies show that ICI treatment has a better therapeutic effect and lesser toxicity in TNBC [24, 25]. Moreover, the vascular endothelial growth factor (VEGF) is an important regulator of tumor angiogenesis and metastasis [26, 27]. Bevacizumab (B) is a recombinant human monoclonal antibody against VEGF that plays various roles in the tumor blood vessels by specifically binding to VEGF and blocking its interaction with receptors [28]. Relevant studies have reported that adding B based on chemotherapeutic drugs can improve the pCR. Antivascular therapy combined with immunotherapy showed an excellent antitumor activity of different cancers [29, 30]. Liu et al*.* showed that antiangiogenic therapy can improve the sensitivity of PD-L1 expression and the infiltration of PD-1/PD-L1 immunotherapy, playing a synergistic sensitization effect and improving the disease-free survival (DFS) and overall survival (OS) of patients with TNBC [31, 32].

Although numerous NCT regimens are currently being used for TNBC, the clinical efficacy of different treatment regimens, especially in terms of long-term survival, remains unclear. Therefore, we conducted a Bayesian meta-analysis of randomized controlled trials (RCTs) to evaluate the effectiveness of different treatment regimens (long-term survival and pCR), thereby providing evidence-based medical information on NCT for TNBC in clinical practice.

## Methods

### Search strategy

This network meta-analysis (NMA) was performed according to the preferred reporting items for systematic reviews and meta-analyses statement [33]. PubMed, EMBASE, Medline, Cochrane Library, Web of Science, main oncology conference of American Society of Clinical Oncology, the European Society of Medical Oncology, and San Antonio Breast Cancer Symposium databases were searched for high-quality RCTs from January 2000 to June 2023. The search was performed using the following keywords without any restrictions: (triple-negative breast cancer OR triple negative breast neoplasm OR er-negative pr-negative her2-negative breast cancer OR TNBC) AND (neoadjuvant therapy OR neoadjuvant treatment OR neoadjuvant chemotherapy OR neoadjuvant chemotherapy treatment) AND (DFS OR disease free survival) AND (OS OR overall survival) AND (pCR OR pathological complete response). The reference lists of relevant studies, reviews, and meta-analyses were manually screened for potentially eligible publications.

### Selection criteria

Eligible trials included those that prospectively compared at least two arms of different neoadjuvant chemotherapeutic regimens in patients with TNBC. Inclusion criteria were as follows: patients with pathologically confirmed TNBC; those with clinical stages of II and III (T1c, N1-2 or T2-4, and N0-2); and those who did not receive surgical NCT. The study end-points included event-free survival (EFS) or DFS, OS, and pCR. The exclusion criteria were as follows: studies involving patients with metastatic TNBC; non-RCTs; articles not written in English; and studies with no data regarding EFS or DFS, OS, and pCR. If several publications from the same trial were identified, only the most recent or complete publications were included.

### Data extraction

Eight reviewers were divided into four groups to independently screen the articles (ZL and JL, FZ and QX, DR and ZL, and YC and SH), perform data extraction (ZL and JL and LZ and ZY), and assess the risk of bias (ZL and JL and LZ and MW). Disagreements were resolved by discussion, with assistance from a third party (GS or JZ) if necessary. The following information was recorded: study, author–year, journal, country, arms, medicine, clinical stage, trial phase, TNBC definition, sample size, and study outcomes (EFS or DFS, OS, and pCR).

### Explanation of treatment regimens and outcome definitions

Currently, the standard treatment options for TNBC are not yet established, and NCT with anthracycline and purple line represents the cornerstone historical standard for TNBC treatment [34]. Our study defined the combination of anthracyclines and taxanes as the preferred treatment option. On this basis, any addition of other therapeutic drugs is a new treatment option.

### Statistical analysis

Hazards ratio (HR) and odds ratio (OR) were used to estimate pooling effect sizes. For pairwise meta-analysis, the Cochrane Q statistic and the I^2^ test were used to calculate heterogeneity. Statistical heterogeneity was defined as P of < 0.1 and/or I^2^ of > 50%. A pairwise meta-analysis was performed using a random-effects model or a fixed-effect model depending on the presence of statistical heterogeneity. All pairwise meta-analyses were performed using the Review Manager version 5.3. Results are reported as HR, OR, and corresponding 95% confidence intervals (CIs). All *P*-values were two sided, and differences with *P* < 0.05 were considered statistically significant. A Bayesian NMA was performed using the Aggregate Data Drug Information System version 1.16.6 (http://www.drugis.org). Node splitting analyses were performed to verify the consistency between direct and indirect evidence. If no significant inconsistency was detected, a consistency model was used to analyze the relative effects of the interventions. Otherwise, an inconsistency model was applied. The “gemtc” package of the R (v14.1) software was used for sorting chats and analyze the data. The NMA results are presented as HR and its corresponding 95% CIs. The “network” packages of the Stata (v14.2) software were used for sorting chats and data analysis. The NMA results are presented as OR and corresponding 95% CIs. The rank probability for each treatment was calculated to determine the treatment ranking. When assessing the merit of the drug efficacy, the surface under the cumulative ranking curve (SUCRA) values was used. It has a value of 0 to 1, and higher SUCRA values indicate better efficacy of the agent.

## Results

### Study selection and characteristics of the included studies

**Fig. 1:**
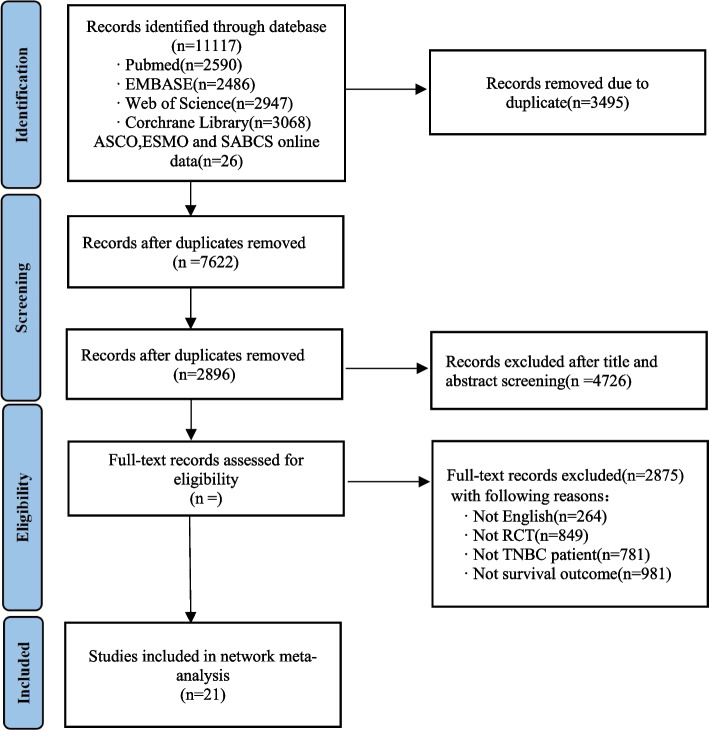
A flowchart of the study selection process

**Fig. 2:**
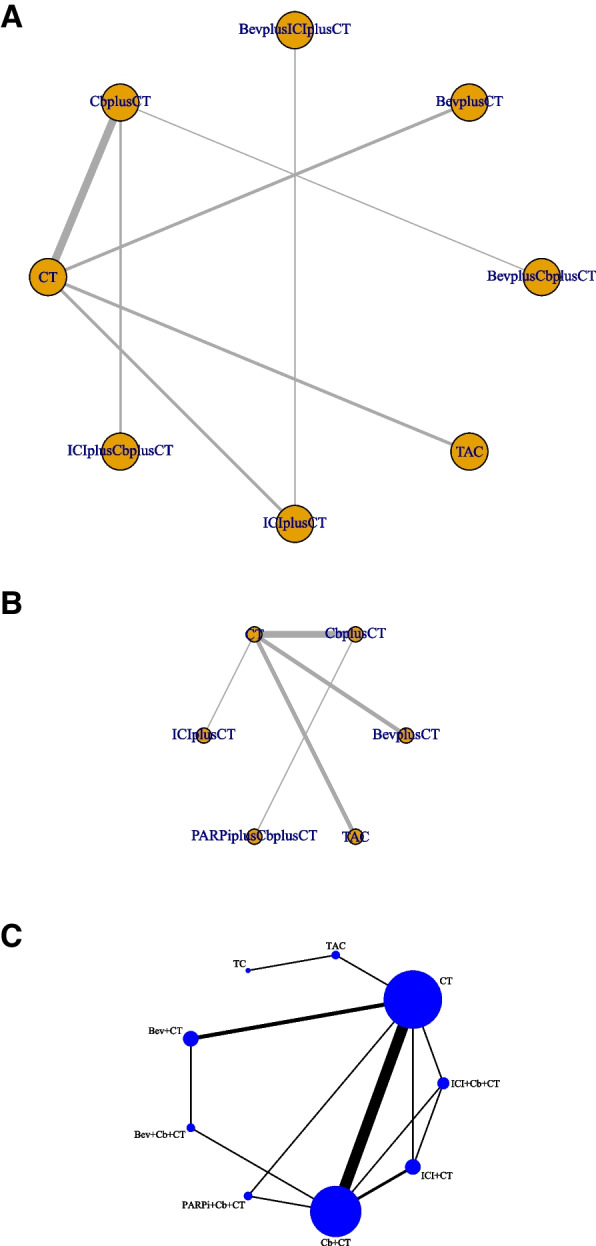
Network plots for eligible comparisons were included in the network meta-analysis. **A** Network diagram of the disease-free survival (DFS). **B** Network diagram of the overall survival (OS). **C** Network diagram of the pathological complete response (pCR)

Figure 1 illustrates the study retrieval process. A total of 10,000 results were obtained from the database, and 1500 studies were automatically removed by Zotero. Based on titles and abstracts, 120 suitable full-text studies were screened, and 31 studies were excluded due to the lack of assessment results. Ultimately, 21 studies involving 8873 patients were included in our reticulated meta-analysis [16–19, 25, 34–49]. Table 1 summarizes the characteristics of the included RCTs. A total of 18 phase III trials and 3 phase II trials were identified. This study evaluated nine treatment regimens in the form of network maps: standard chemotherapeutic agents, TAC (taxanes, anthracycline, and cyclophosphamide), TC (taxanes and cyclophosphamide), B, P, B + P, P + PARPi, ICI, and ICI + P (Fig. 2).

**Table 1:** Main characteristics of the included clinical studies

Study ID	Auther-year	Journal	Masking	RCT	Arms	Medicine	Clinical stage	TNBC definition	Endpoints	Total numbers	pCR (%)	HR for DFS	*P* value	HR for OS	*P* value
NATT trial	Xiaosong Chen-2013	Breast Cancer Res Treat	Open-label	RCT	2	TAC vs TC	IIB—IIIC	ER < 1% PR < 1% Her2(0–2 + FISH-)	pCR EFS DFS OS	26	15.40%	DFS 0.33(0.12–0.95)	0.004	0.52(0.06–0.46)	0.045
										23	4.30%				
PREPARE trial(NCT00544232)	M. Untch-2011	Annals of Oncology	Open-label	RCT	2	ddE-ddT-CMF vs EC-T	IIB—IIIC	ER < 1% PR < 1% Her2(0–2 + FISH-)	pCR EFS DFS OS	363	18.70%	1.14(0.85–1.52)	0.37	1.26(0.86–1.85)	0.237
										370	13.20%				
NeoSTOP(NCT02413320)	Priyanka Sharma-2021	Clin Cancer Res	Open-label	RCT	2	CbP-AC vs CbD	I—III	ER < 10% PR < 10% Her2(-)	pCR EFS OS	48	54.00%	EFS → DFS 2.85(0.34–23.60)	> 0.05	1.83(0.28–11.76)	> 0.05
										52	54.00%				
NeoCART (NCT03154749)	Liulu Zhang-2021	Int J Cancer	Open-label	RCT	2	**DCb vs EC-D**	II-III	ER < 1% PR < 1% Her2(0–2 + FISH-)	pCR EFS OS	44	61.40%	EFS → DFS 0.76(0.2–2.84)	0.683	0.96(0.19–4.76)	0.959
										44	38.60%				
(UMIN000003355)	Madoka Iwase-2020	Breast Cancer Research and Treatment	Open-label	RCT	2	**CbP-CEF vs P-CEF**	II-III	ER(-) PR(-) Her2(0–2 + FISH-)	DFS OS pCR	37	61.20%	0.22(0.06–0.82)	0.015	0.12(0.01–0.96)	0.046
										38	26.30%				
GeparOcto (NCT02125344)	Andreas Schneeweiss-2022	European Journal of Cancer	Open-label	RCT	2	**PMCb vs iddEPC**	II	ER(-) PR(-) Her2(0–2 + FISH-)	pCR iDFS OS	203	48.00%	0.73(0.47–1.13)	0.1562	0.66(0.38–1.15)	0.1442
										200	48.30%				
WSG-ADAPT-TN (NCT01815242)	Oleg Gluz-2022	European Journal of Cancer	Open-label	RCT	2	**nab-pac + Cb vs** **nab-pac + G**	I-III	ER < 1% PR < 1% Her2(0–2 + FISH-)	pCR i/dDFS OS	182	44.00%	1.21(0.76–1.94)	0.424	1.06(0.63–1.78)	0.836
										154	28.00%				
GeparSixto (NCT01426880)	Eric Hahnen-2017	JAMA Oncol	Open-label	RCT	2	PMBevCb vs PMBev	II-III	ER < 1% PR < 1% Her2(0–2 + FISH-)	pCR DFS	158	53.20%	0.53(0.29–0.96)	0.04	NA	
										157	36.90%				
(NCT01276769)	Pin Zhang-2016	Oncotarget	Open-label	RCT	2	**pac + Cb vs pacE**	II-III	ER < 10% PR < 10% Her2(0–2 + FISH-)	pCR RFS OS	47	38.60%	(RFS → DFS) 0.35(0.13–0.96)	0.043	1.20(0.37–3.87)	0.350
										44	14.00%				
CALGB 40603 (NCT00861705)	William M.Sikov-2022	J Clin Oncol	Open-label	RCT	2	**pac + Cb + AC vs pac + AC**	II-III	ER < 10% PR < 10% Her2(-)	pCR EFS OS	221	54%	(EFS → DFS) 0.94(0.67–1.32)	0.7210	OS 1.12(0.77–1.61)	0.5585
										212	41%				
(ChiCTR-TRC-14005019)	Wenting Yan-2022	Ther Adv Med Oncol	Open-label	RCT	2	**TEL vs TE**	I-III	ER < 10% PR < 10% Her2(0–2 + FISH-)	tpCR DFS OS	99	41.40%	0.44(0.21–0.90)	0.028	0.44(0.18–1.02)	0.061
										101	17.80%				
Kun Wang (SABCS)	Kun Wang-2022	SABCS	Open-label	RCT	2	**wPCb-AC vs wP-AC**	II	NA	DFS OS pCR	365	55.20%	0.79(0.61–1.02)	0.073	0.75(0.57–0.98)	0.034
										355	41.50%				
I-SPY2 Trial(NCT01042379)	Nanda, R-2020	JAMA Oncol	Open-label	RCT	2	P + pac + AC vs PBO + pac + AC	II-III	ER(-) PR(-) Her2(-)	pCR EFS	29	60.00%	EFS → DFS 0.6(0.36–3.81)	> 0.05	NA	NA
										85	22.00%				
										168	41.1%				
KEYNOTE-173 (NCT02622074)	P. Schmid-2020	Annals of Oncology	Open-label	RCT	6	P + Nab-pac + AC vs P + Nab-pac + Cb + AC vs P + Nab-pac + Cb + AC vs P + Nab-pac + Cb + AC vs P + pac + Cb + AC vs P + pac + Cb + AC	II-III	ER < 1% PR < 1% Her2(0–2 + FISH-)	pCR EFS OS	10	60%	EFS → DFS NA	NA	NA	NA
										10	80%				
										10	80%				
										10	60%				
										10	30%				
										10	50%				
KEYNOTE-522(NCT03036488)	P. Schmid-2020	New England Journal of Medicine	Open-label	RCT	2	P + pac + Cb + A/EC vs pbo + pac + Cb + A/EC	II-III	ER(-) PR(-) Her2(0–2 + FISH-)	pCR EFS	784	64.80%	EFS → DFS 0.63(0.48–0.82)	*P* < 0.001	NA	
										390	51.20%				
GeparNuevo (NCT02685059)	S. Loibl-2022	Annals of Oncology	Open-label	RCT	2	Dur + nab-pac vs PBO + nab-pac	I-III	NA	pCR iDFS DDFS OS	88	NA	DDFS → DFS 0.31(0.13–0.74)	0.005	0.24(0.08–0.72)	0.006
										86	NA				
BrighTNess (NCT02032277)	Sibylle Loibl-2022	Annals of Oncology	Open-label	RCT	3	Paclitaxel + carboplatin + veliparib vs Paclitaxel + carboplatin + veliparib placebo vs Paclitaxel + carboplatin placebo + veliparib placebo	I-III	ER < 1% PR < 1% Her2(0 or 1 +)	pCR EFS OS breast-conservation surgery	316	53.00%	EFS → DFS 1.12(0.72–1.72) (1 vs 2) 0.56(0.34–0.93) (2 vs 3)	0.62(1 vs 2) 0.02(2 vs 3)	1.25 (0.70–2.24) (1 vs 2) 0.63 (0.33–1.21) (2 vs 3)	0.46 (1 vs 2) 0.17 (2 vs 3)
										160	58.00%				
										158	31.00%				
NSABP-B40(NCT00408408)	Harry D Bear-2015	Lancet Oncol	Open-label	RCT	2	Bev + vs Non-Bev	I-III	ER(-) PR(-) Her2(-)	pCR EFS	592	51.10%	0.8 (0.63–1.01)	0.06	0.65(0.49–0.88)	0.004
										594	47.10%				
GeparQuinto(NCT00567554)	G. von Minckwitz-2014	Annals of Oncology	Open-label	RCT	2	ECB-DB vs EC-D	II-III	ER (< 10%) PR (< 10%) Her2(0–2 + FISH-)	pCR DFS OS	323	39.30%	1.03(0.84–1.25)	0.784	0.97(0.75–1.26)	0.842
										340	27.90%				
SWOG S0800(NCT00856492)	Z. A. Nahleh-2016	Breast Cancer Res Treat	Open-label	RCT	2	Bev + nab-pac + ddAC vs Non-Bev-based	IIB—IIIC	ER(-) PR(-) Her2(-)	pCR DFS OS	98	59.40%	EFS → DFS 0.46(0.2–1.05)	0.06	0.49(0.19–1.29)	0.14
										113	28.60%				
ARTemis(NCT01093235)	H. M. Earl-2017	Annals of Oncology	Open-label	RCT	2	Bev + D-FEC vs D-FEC	NA	(ER) status as negative when Allred score was 0–2/8; ER weakly positive was 3–5/8; and ER strongly positive was 6–8/8 Her2(0–2 + FISH-)	pCR DFS OS	388	22.00%	1.18(0.89–1.57)	0.25	1.26(0.9–1.76)	0.19
										393	17.00%				
Study ID	Auther-year	Journal	Masking	RCT	Arms	Medicine	Clinical stage	TNBC definition	Endpoints	Total numbers	pCR (%)	HR for DFS	*P* value	HR for OS	*P* value
NATT trial	Xiaosong Chen-2013	Breast Cancer Res Treat	Open-label	RCT	2	TAC vs TC	IIB—IIIC	ER < 1% PR < 1% Her2(0–2 + FISH-)	pCR EFS DFS OS	26	15.40%	DFS 0.33(0.12–0.95)	0.004	0.52(0.06–0.46)	0.045
										23	4.30%				
PREPARE trial(NCT00544232)	M. Untch-2011	Annals of Oncology	Open-label	RCT	2	ddE-ddT-CMF vs EC-T	IIB—IIIC	ER < 1% PR < 1% Her2(0–2 + FISH-)	pCR EFS DFS OS	363	18.70%	1.14(0.85–1.52)	0.37	1.26(0.86–1.85)	0.237
										370	13.20%				
NeoSTOP(NCT02413320)	Priyanka Sharma-2021	Clin Cancer Res	Open-label	RCT	2	CbP-AC vs CbD	I—III	ER < 10% PR < 10% Her2(-)	pCR EFS OS	48	54.00%	EFS → DFS 2.85(0.34–23.60)	> 0.05	1.83(0.28–11.76)	> 0.05
										52	54.00%				
NeoCART (NCT03154749)	Liulu Zhang-2021	Int J Cancer	Open-label	RCT	2	**DCb vs EC-D**	II-III	ER < 1% PR < 1% Her2(0–2 + FISH-)	pCR EFS OS	44	61.40%	EFS → DFS 0.76(0.2–2.84)	0.683	0.96(0.19–4.76)	0.959
										44	38.60%				
(UMIN000003355)	Madoka Iwase-2020	Breast Cancer Research and Treatment	Open-label	RCT	2	**CbP-CEF vs P-CEF**	II-III	ER(-) PR(-) Her2(0–2 + FISH-)	DFS OS pCR	37	61.20%	0.22(0.06–0.82)	0.015	0.12(0.01–0.96)	0.046
										38	26.30%				
GeparOcto (NCT02125344)	Andreas Schneeweiss-2022	European Journal of Cancer	Open-label	RCT	2	**PMCb vs iddEPC**	II	ER(-) PR(-) Her2(0–2 + FISH-)	pCR iDFS OS	203	48.00%	0.73(0.47–1.13)	0.1562	0.66(0.38–1.15)	0.1442
										200	48.30%				
WSG-ADAPT-TN (NCT01815242)	Oleg Gluz-2022	European Journal of Cancer	Open-label	RCT	2	**nab-pac + Cb vs** **nab-pac + G**	I-III	ER < 1% PR < 1% Her2(0–2 + FISH-)	pCR i/dDFS OS	182	44.00%	1.21(0.76–1.94)	0.424	1.06(0.63–1.78)	0.836
										154	28.00%				
GeparSixto (NCT01426880)	Eric Hahnen-2017	JAMA Oncol	Open-label	RCT	2	PMBevCb vs PMBev	II-III	ER < 1% PR < 1% Her2(0–2 + FISH-)	pCR DFS	158	53.20%	0.53(0.29–0.96)	0.04	NA	
										157	36.90%				
(NCT01276769)	Pin Zhang-2016	Oncotarget	Open-label	RCT	2	**pac + Cb vs pacE**	II-III	ER < 10% PR < 10% Her2(0–2 + FISH-)	pCR RFS OS	47	38.60%	(RFS → DFS) 0.35(0.13–0.96)	0.043	1.20(0.37–3.87)	0.350
										44	14.00%				
CALGB 40603 (NCT00861705)	William M.Sikov-2022	J Clin Oncol	Open-label	RCT	2	**pac + Cb + AC vs pac + AC**	II-III	ER < 10% PR < 10% Her2(-)	pCR EFS OS	221	54%	(EFS → DFS) 0.94(0.67–1.32)	0.7210	OS 1.12(0.77–1.61)	0.5585
										212	41%				
(ChiCTR-TRC-14005019)	Wenting Yan-2022	Ther Adv Med Oncol	Open-label	RCT	2	**TEL vs TE**	I-III	ER < 10% PR < 10% Her2(0–2 + FISH-)	tpCR DFS OS	99	41.40%	0.44(0.21–0.90)	0.028	0.44(0.18–1.02)	0.061
										101	17.80%				
Kun Wang (SABCS)	Kun Wang-2022	SABCS	Open-label	RCT	2	**wPCb-AC vs wP-AC**	II	NA	DFS OS pCR	365	55.20%	0.79(0.61–1.02)	0.073	0.75(0.57–0.98)	0.034
										355	41.50%				
I-SPY2 Trial(NCT01042379)	Nanda, R-2020	JAMA Oncol	Open-label	RCT	2	P + pac + AC vs PBO + pac + AC	II-III	ER(-) PR(-) Her2(-)	pCR EFS	29	60.00%	EFS → DFS 0.6(0.36–3.81)	> 0.05	NA	NA
										85	22.00%				
										168	41.1%				
KEYNOTE-173 (NCT02622074)	P. Schmid-2020	Annals of Oncology	Open-label	RCT	6	P + Nab-pac + AC vs P + Nab-pac + Cb + AC vs P + Nab-pac + Cb + AC vs P + Nab-pac + Cb + AC vs P + pac + Cb + AC vs P + pac + Cb + AC	II-III	ER < 1% PR < 1% Her2(0–2 + FISH-)	pCR EFS OS	10	60%	EFS → DFS NA	NA	NA	NA
										10	80%				
										10	80%				
										10	60%				
										10	30%				
										10	50%				
KEYNOTE-522(NCT03036488)	P. Schmid-2020	New England Journal of Medicine	Open-label	RCT	2	P + pac + Cb + A/EC vs pbo + pac + Cb + A/EC	II-III	ER(-) PR(-) Her2(0–2 + FISH-)	pCR EFS	784	64.80%	EFS → DFS 0.63(0.48–0.82)	*P* < 0.001	NA	
										390	51.20%				
GeparNuevo (NCT02685059)	S. Loibl-2022	Annals of Oncology	Open-label	RCT	2	Dur + nab-pac vs PBO + nab-pac	I-III	NA	pCR iDFS DDFS OS	88	NA	DDFS → DFS 0.31(0.13–0.74)	0.005	0.24(0.08–0.72)	0.006
										86	NA				
BrighTNess (NCT02032277)	Sibylle Loibl-2022	Annals of Oncology	Open-label	RCT	3	Paclitaxel + carboplatin + veliparib vs Paclitaxel + carboplatin + veliparib placebo vs Paclitaxel + carboplatin placebo + veliparib placebo	I-III	ER < 1% PR < 1% Her2(0 or 1 +)	pCR EFS OS breast-conservation surgery	316	53.00%	EFS → DFS 1.12(0.72–1.72) (1 vs 2) 0.56(0.34–0.93) (2 vs 3)	0.62(1 vs 2) 0.02(2 vs 3)	1.25 (0.70–2.24) (1 vs 2) 0.63 (0.33–1.21) (2 vs 3)	0.46 (1 vs 2) 0.17 (2 vs 3)
										160	58.00%				
										158	31.00%				
NSABP-B40(NCT00408408)	Harry D Bear-2015	Lancet Oncol	Open-label	RCT	2	Bev + vs Non-Bev	I-III	ER(-) PR(-) Her2(-)	pCR EFS	592	51.10%	0.8 (0.63–1.01)	0.06	0.65(0.49–0.88)	0.004
										594	47.10%				
GeparQuinto(NCT00567554)	G. von Minckwitz-2014	Annals of Oncology	Open-label	RCT	2	ECB-DB vs EC-D	II-III	ER (< 10%) PR (< 10%) Her2(0–2 + FISH-)	pCR DFS OS	323	39.30%	1.03(0.84–1.25)	0.784	0.97(0.75–1.26)	0.842
										340	27.90%				
SWOG S0800(NCT00856492)	Z. A. Nahleh-2016	Breast Cancer Res Treat	Open-label	RCT	2	Bev + nab-pac + ddAC vs Non-Bev-based	IIB—IIIC	ER(-) PR(-) Her2(-)	pCR DFS OS	98	59.40%	EFS → DFS 0.46(0.2–1.05)	0.06	0.49(0.19–1.29)	0.14
										113	28.60%				
ARTemis(NCT01093235)	H. M. Earl-2017	Annals of Oncology	Open-label	RCT	2	Bev + D-FEC vs D-FEC	NA	(ER) status as negative when Allred score was 0–2/8; ER weakly positive was 3–5/8; and ER strongly positive was 6–8/8 Her2(0–2 + FISH-)	pCR DFS OS	388	22.00%	1.18(0.89–1.57)	0.25	1.26(0.9–1.76)	0.19
										393	17.00%				
Study ID	Auther-year	Journal	Masking	RCT	Arms	Medicine	Clinical stage	TNBC definition	Endpoints	Total numbers	pCR (%)	HR for DFS	*P* value	HR for OS	*P* value
NATT trial	Xiaosong Chen-2013	Breast Cancer Res Treat	Open-label	RCT	2	TAC vs TC	IIB—IIIC	ER < 1% PR < 1% Her2(0–2 + FISH-)	pCR EFS DFS OS	26	15.40%	DFS 0.33(0.12–0.95)	0.004	0.52(0.06–0.46)	0.045
										23	4.30%				
PREPARE trial(NCT00544232)	M. Untch-2011	Annals of Oncology	Open-label	RCT	2	ddE-ddT-CMF vs EC-T	IIB—IIIC	ER < 1% PR < 1% Her2(0–2 + FISH-)	pCR EFS DFS OS	363	18.70%	1.14(0.85–1.52)	0.37	1.26(0.86–1.85)	0.237
										370	13.20%				
NeoSTOP(NCT02413320)	Priyanka Sharma-2021	Clin Cancer Res	Open-label	RCT	2	CbP-AC vs CbD	I—III	ER < 10% PR < 10% Her2(-)	pCR EFS OS	48	54.00%	EFS → DFS 2.85(0.34–23.60)	> 0.05	1.83(0.28–11.76)	> 0.05
										52	54.00%				
NeoCART (NCT03154749)	Liulu Zhang-2021	Int J Cancer	Open-label	RCT	2	DCb vs EC-D	II-III	ER < 1% PR < 1% Her2(0–2 + FISH-)	pCR EFS OS	44	61.40%	EFS → DFS 0.76(0.2–2.84)	0.683	0.96(0.19–4.76)	0.959
										44	38.60%				
(UMIN000003355)	Madoka Iwase-2020	Breast Cancer Research and Treatment	Open-label	RCT	2	CbP-CEF vs P-CEF	II-III	ER(-) PR(-) Her2(0–2 + FISH-)	DFS OS pCR	37	61.20%	0.22(0.06–0.82)	0.015	0.12(0.01–0.96)	0.046
										38	26.30%				
GeparOcto (NCT02125344)	Andreas Schneeweiss-2022	European Journal of Cancer	Open-label	RCT	2	PMCb vs iddEPC	II	ER(-) PR(-) Her2(0–2 + FISH-)	pCR iDFS OS	203	48.00%	0.73(0.47–1.13)	0.1562	0.66(0.38–1.15)	0.1442
										200	48.30%				
WSG-ADAPT-TN (NCT01815242)	Oleg Gluz-2022	European Journal of Cancer	Open-label	RCT	2	nab-pac + Cb vs nab-pac + G	I-III	ER < 1% PR < 1% Her2(0–2 + FISH-)	pCR i/dDFS OS	182	44.00%	1.21(0.76–1.94)	0.424	1.06(0.63–1.78)	0.836
										154	28.00%				
GeparSixto (NCT01426880)	Eric Hahnen-2017	JAMA Oncol	Open-label	RCT	2	PMBevCb vs PMBev	II-III	ER < 1% PR < 1% Her2(0–2 + FISH-)	pCR DFS	158	53.20%	0.53(0.29–0.96)	0.04	NA	
										157	36.90%				
(NCT01276769)	Pin Zhang-2016	Oncotarget	Open-label	RCT	2	pac + Cb vs pacE	II-III	ER < 10% PR < 10% Her2(0–2 + FISH-)	pCR RFS OS	47	38.60%	(RFS → DFS) 0.35(0.13–0.96)	0.043	1.20(0.37–3.87)	0.350
										44	14.00%				
CALGB 40603 (NCT00861705)	William M.Sikov-2022	J Clin Oncol	Open-label	RCT	2	pac + Cb + AC vs pac + AC	II-III	ER < 10% PR < 10% Her2(-)	pCR EFS OS	221	54%	(EFS → DFS) 0.94(0.67–1.32)	0.7210	OS 1.12(0.77–1.61)	0.5585
										212	41%				
(ChiCTR-TRC-14005019)	Wenting Yan-2022	Ther Adv Med Oncol	Open-label	RCT	2	TEL vs TE	I-III	ER < 10% PR < 10% Her2(0–2 + FISH-)	tpCR DFS OS	99	41.40%	0.44(0.21–0.90)	0.028	0.44(0.18–1.02)	0.061
										101	17.80%				
Kun Wang (SABCS)	Kun Wang-2022	SABCS	Open-label	RCT	2	wPCb-AC vs wP-AC	II	NA	DFS OS pCR	365	55.20%	0.79(0.61–1.02)	0.073	0.75(0.57–0.98)	0.034
										355	41.50%				
I-SPY2 Trial(NCT01042379)	Nanda, R-2020	JAMA Oncol	Open-label	RCT	2	P + pac + AC vs PBO + pac + AC	II-III	ER(-) PR(-) Her2(-)	pCR EFS	29	60.00%	EFS → DFS 0.6(0.36–3.81)	> 0.05	NA	NA
										85	22.00%				
										168	41.1%				
KEYNOTE-173(NCT02622074)	P. Schmid-2020	Annals of Oncology	Open-label	RCT	6	P + Nab-pac + AC vs P + Nab-pac + Cb + AC vs P + Nab-pac + Cb + AC vs P + Nab-pac + Cb + AC vs P + pac + Cb + AC vs P + pac + Cb + AC	II-III	ER < 1% PR < 1% Her2(0–2 + FISH-)	pCR EFS OS	10	60%	EFS → DFS NA	NA	NA	NA
										10	80%				
										10	80%				
										10	60%				
										10	30%				
										10	50%				
KEYNOTE-522(NCT03036488)	P. Schmid-2020	New England Journal of Medicine	Open-label	RCT	2	P + pac + Cb + A/EC vs pbo + pac + Cb + A/EC	II-III	ER(-) PR(-) Her2(0–2 + FISH-)	pCR EFS	784	64.80%	EFS → DFS 0.63(0.48–0.82)	*P* < 0.001	NA	
										390	51.20%				
GeparNuevo (NCT02685059)	S. Loibl-2022	Annals of Oncology	Open-label	RCT	2	Dur + nab-pac vs PBO + nab-pac	I-III	NA	pCR iDFS DDFS OS	88	NA	DDFS → DFS 0.31(0.13–0.74)	0.005	0.24(0.08–0.72)	0.006
										86	NA				
BrighTNess(NCT02032277)	Sibylle Loibl-2022	Annals of Oncology	Open-label	RCT	3	Paclitaxel + carboplatin + veliparib vs Paclitaxel + carboplatin + veliparib placebo vs Paclitaxel + carboplatin placebo + veliparib placebo	I-III	ER < 1% PR < 1% Her2(0 or 1 +)	pCR EFS OS breast-conservation surgery	316	53.00%	EFS → DFS 1.12(0.72–1.72) (1 vs 2) 0.56(0.34–0.93) (2 vs 3)	0.62(1 vs 2) 0.02(2 vs 3)	1.25 (0.70–2.24) (1 vs 2) 0.63 (0.33–1.21) (2 vs 3)	0.46 (1 vs 2) 0.17 (2 vs 3)
										160	58.00%				
										158	31.00%				
NSABP-B40(NCT00408408)	Harry D Bear-2015	Lancet Oncol	Open-label	RCT	2	Bev + vs Non-Bev	I-III	ER(-) PR(-) Her2(-)	pCR EFS	592	51.10%	0.8(0.63–1.01)	0.06	0.65(0.49–0.88)	0.004
										594	47.10%				
GeparQuinto(NCT00567554)	G. von Minckwitz-2014	Annals of Oncology	Open-label	RCT	2	ECB-DB vs EC-D	II-III	ER (< 10%) PR (< 10%) Her2(0–2 + FISH-)	pCR DFS OS	323	39.30%	1.03(0.84–1.25)	0.784	0.97(0.75–1.26)	0.842
										340	27.90%				
SWOG S0800(NCT00856492)	Z. A. Nahleh-2016	Breast Cancer Res Treat	Open-label	RCT	2	Bev + nab-pac + ddAC vs Non-Bev-based	IIB—IIIC	ER(-) PR(-) Her2(-)	pCR DFS OS	98	59.40%	EFS → DFS 0.46(0.2–1.05)	0.06	0.49(0.19–1.29)	0.14
										113	28.60%				
ARTemis(NCT01093235)	H. M. Earl-2017	Annals of Oncology	Open-label	RCT	2	Bev + D-FEC vs D-FEC	NA	(ER) status as negative when Allred score was 0–2/8; ER weakly positive was 3–5/8; and ER strongly positive was 6–8/8 Her2(0–2 + FISH-)	pCR DFS OS	388	22.00%	1.18(0.89–1.57)	0.25	1.26(0.9–1.76)	0.19
										393	17.00%

### DFS

**Fig. 3:**
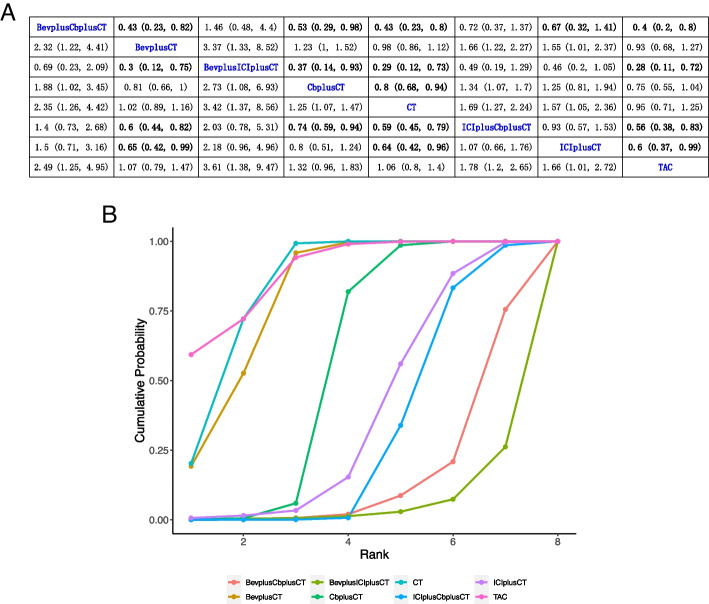
Bayesian network meta-analysis for disease-free survival (DFS). **A** League comparison table. Data are expressed as hazards ratio (HR) and 95% confidence interval (CI). HR of < 1 supports column definition processing, whereas HR of > 1 supports row definition processing. **B** Plot of sequencing probabilities for nine DFS schemes. The larger the area of the curve and the X-axis, the higher the recommended treatment

Of the 21 studies, 20 reported data on DFS, with 3 studies including standard chemotherapy, 8 studies including P-containing regimen, 1 study including B + P-containing regimen, 4 studies including B-containing regimen, 1 study including P + PARPi-containing regimen, 2 studies including ICI-containing regimen, and 1 study including ICI + P-containing regimen, all of which were NCTs. Results showed that CT compared with P (HR, 0.8; 95% CI, 0.68–0.94), B + ICI (HR, 0.29; 95% CI, 0.12–0.73), and B + P (HR, 0.43; 95% CI, 0.23–0.8) had a significant benefit of DFS. Figure 3A summarizes the results of DFS analysis.

A cumulative ranking of the nine treatment regimens was also analyzed. The results showed that TAC (89.23%), CT (84.53%), B (81.06%), and P (55,30%) ranked first to forth, while ICI (37.86%), ICI + P (30.94%), B + P (15.48%), and B + ICI (5.58%) ranked fifth to eighth (Fig. 3B).

### OS

**Fig. 4:**
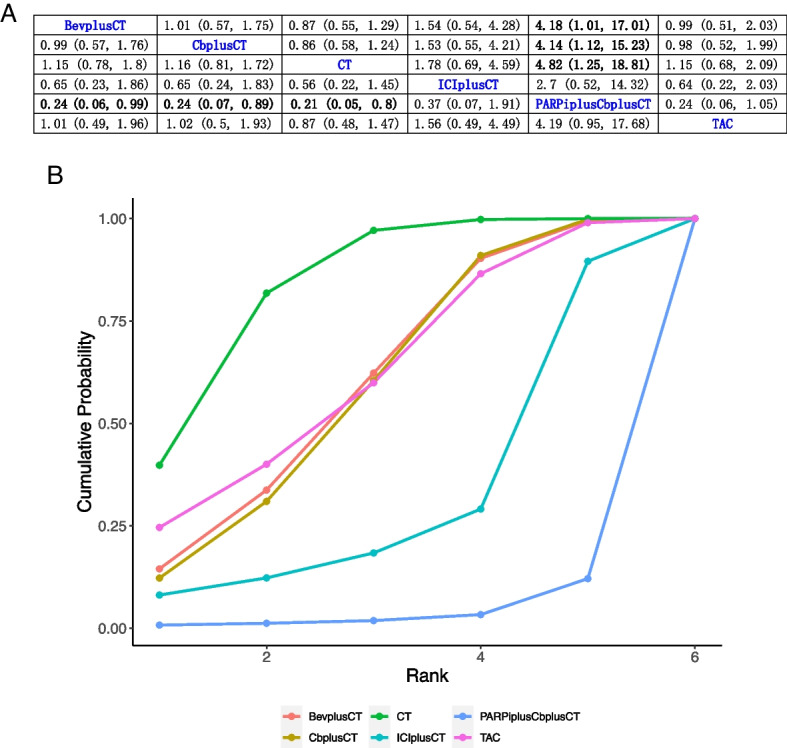
Bayesian network meta-analysis of the overall survival (OS). **A** League comparison table. Data are expressed as hazards ratio (HR) and 95% confidence interval (CI). HR of < 1 supports the column definition processing, whereas HR of > 1 supports the row definition processing. **B** Plot of sequencing probabilities for nine OS schemes. The larger the area of the curve and the X-axis, the higher the recommended treatment

Of the 21 studies, 17 reported data on OS, with 3 studies including B-containing regimen, 3 studies including standard chemotherapy, 8 studies including P-containing regimen, 2 studies including ICI-containing regimen, and 1 study including PARPi + P-containing regimen, all of which were NCTs. Results showed that PARPi + P-containing regimen compared with B (HR, 0.24; 95% CI, 0.06–0.99), P (HR, 0.24; 95% CI, 0.07–0.89), and standard chemotherapy (HR, 0.21; 95% CI, 0.05–0.8) had a significant benefit of OS. Figure 4A summarizes the results of OS analysis.

A cumulative ranking of the nine treatment regimens was also analyzed. The results showed that CT (83.70%), TAC (62.02%), and B-containing regimens (60.06%) ranked first to third, while P-containing regimens (58.89%), ICI-containing regimens (31.48%), and PARPi + P-containing regimens (3.85%) ranked forth to sixth (Fig. 4B).

### pCR

**Fig. 5:**
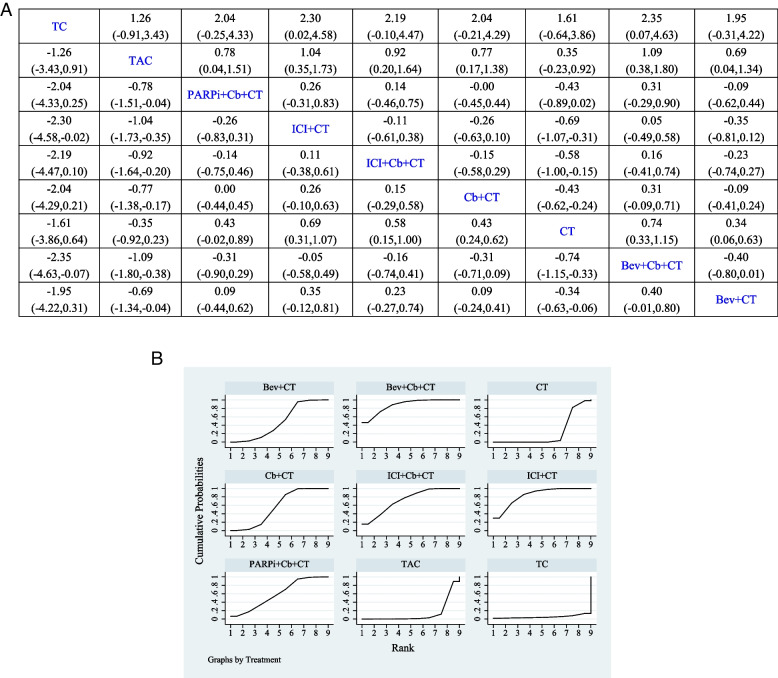
Bayesian network meta-analysis of pathological complete response (pCR). **A** The league table of comparisons. Data are presented as odds radio (OR) and 95% confidence intervals (CI). An OR of > 1 favors the column-defining treatment, and an OR of < 1 favors the row-defining treatment. **B** Cumulative sequence diagram of nine pCR schemes. The higher the SUCRA value, the higher the ranking

All 21 included trials reported pCR, with 3 studies including standard chemotherapy, 8 studies including P-containing regimen, 1study including B + P-containing regimen, 4 studies including B-containing regimen, 1 study including P + PARPi-containing regimen, 2 studies including ICI-containing regimen, and 2 studies including ICI + P-containing regimen, all of which were NCTs. The incidence of pCR in the PARPi + P-containing regimen (OR, 0.43; 95% CI, − 0.02 to 0.89), P-containing regimen (OR, 0.43; 95% CI, 0.24–0.62), and B-containing regimen (OR, 0.34; 95% CI, 0.06–0.63) was significantly higher than that of standard chemotherapeutic agents. Figure 5A summarizes the results of pCR analysis.

A cumulative ranking of the nine treatment regimens was also analyzed. The results showed that B + P-containing regimens (82.7%), ICI + P-containing regimens (80.2%), ICI-containing regimens (61.8%), and P-containing regimens (55.0%) ranked first to forth, while PARPi + P-containing regimens (53.5%), B-containing regimens (44.4%), CT (20.5%), TAC (1.8%), and TC (1.5%) ranked fifth to ninth (Fig. 5B).

## Discussion

Currently, the combination of P, B, PARPi, and ICI based on anthracyclines, cyclophosphamides, and taxanes has paved a new avenue for TNBC treatment [50–54]. However, the long-term survival after neoadjuvant treatment in patients with TNBC under different treatment regimens remains unclear. Therefore, we conducted a Bayesian meta-analysis of RCTs to evaluate the effectiveness of different treatment regimens (long-term survival and pCR) and provide evidence-based medical information on NCT for TNBC in clinical practice. The results showed that, based on SUCRA values, standard chemotherapy is still a better choice for long-term survival consideration compared with NCT for TNBC, and the B + P-containing regimen is most likely the optimal NCT option for TNBC based on pCR results.

In 2022, Li et al [53]. published an NMA evaluating eight neoadjuvant treatment options for TNBC. The treatment regimen included the combination of P, B, PARPi, and ICI. In this previous study, the observation indicator was pCR; our study added survival indicators to determine the efficacy ranking of several treatment options for TNBC.

This study included 21 RCTs involving 8873 patients with TNBC. Of these, 20 RCTs reported data on DFS; however, only 7 RCTs reported statistical significance for DFS, with 2 studies using standard chemotherapies, 3 studies using P-containing regimens, 1 using ICI-containing regimens, and 1 trial using B + P-containing regimens. Longer survival was also reported in the remaining 13 trials without significant statistical significance. Due to limited DFS data, we treated data regarding EFS, relapse-free survival, and distant DFS reported in these studies as DFS data; however, the significant DFS data remained somewhat unsatisfactory. It may be related to the small number of patients included in the study or the lack of relevant data. When we summarized 20 studies based on SUCRA values, the proportion of studies using standard chemotherapy was relatively high, and the top three treatment options were standard chemotherapy (89.23%), B-containing regimens (81.06%), and P-containing regimens (55.30%).

In our NMA, 17 of 21 trials reported data on OS, but only 5 of them reported statistical significance for OS, which included 1 study using standard chemotherapy, 2 studies using P-containing regimens, 1 study using ICI-containing regimens, and 1 study using B-containing regimens. Longer survival was also reported in the remaining 12 trials, but without significant statistical significance. This may be related to the small number of patients included in the study or the short follow-up time; however, the addition of P, B, and ICI to the standard chemotherapy can partly prolong the OS of patients with TNBC [55–59]. Further large-scale clinical trials are warranted to confirm their efficacy in the future. In terms of OS, when we summarized 17 studies based on SUCRA values, a high proportion of studies were based on standard chemotherapy, and the top three treatment options were standard chemotherapy (83.70%), B-containing regimens (60.06%), and P-containing regimens (58.89%).

All 21 trials reported pCR data, which were shown to be statistically significant. Compared with standard chemotherapeutic agents alone, P-containing regimens, PARPi-containing regimens, or neoadjuvant regimens based on B or ICI showed significant associations with better pCR. Moreover, a recent paired meta-analysis revealed that NCT based on the above regimens significantly improved pCR in patients with TNBC compared with standard chemotherapy [53], which is consistent with our findings. The results of reticulation analysis based on SUCRA values suggested that B + P-containing regimens are most likely the optimal NCT option for TNBC. The subsequent regimens were ICI + P (80.2%) and ICI (61.8%), and the final recommendation was standard chemotherapy.

This study has some limitations. First, the small number of clinical patients included in these studies or insufficient follow-up time may have caused a bias on the study results. Second, the RCTs included in this study were mainly based on standard chemotherapy, and the proportion of pairs among nine neoadjuvant regimens was small, which may have led to missing indirect contrast data, resulting in inaccurate estimation of the optimal treatment regimen. Third, although we included survival indicators, survival data of different treatment regimens remained insufficient. However, we believe that the use of our carefully pooled data and statistical methods can overcome these limitations of reticulation analysis.

## Conclusions

This NMA demonstrated that standard chemotherapy is a good choice with respect to long-term survival, and B-containing regimens are associated with significantly higher pCR rates among patients with neoadjuvant TNBC. Future research should focus on evaluating larger clinical studies to obtain further survival data to help optimize personalized treatment for patients with TNBC.

## Data Availability

All data generated or analysed during this study are included in this published article.

## Abbreviations

TNBC: Triple-negative breast cancer
NMA: Network meta-analysis
NCT: Neoadjuvant chemotherapy
RCTs: Randomized controlled trials
HR: Hazard ratio
CI: Confidence interval
DFS: Disease-free survival
OS: Overall survival
ORs: Odds ratios
pCR: Pathologic complete response
B: Bevacizumab
P: Platinum
PARPi: Poly-ADP-ribose polymerase inhibitors
ICI: Immune checkpoint inhibitor
SUCRA: Surface under the cumulative ranking curve
PD-1: Programmed death protein 1
PD-L1: Programmed death ligand 1
VEGF: Vascular endothelial growth factor
EFS: Event-free survival

## References

[1] Siegel RL, Miller KD, Fuchs HE (2022). Cancer statistics. CA Cancer J Clin.

[2] Sung H, Ferlay J, Siegel RL (2021). Global Cancer Statistics 2020: GLOBOCAN Estimates of Incidence and Mortality Worldwide for 36 Cancers in 185 Countries. CA A Cancer J Clinicians.

[3] Brenton JD, Carey LA, Ahmed AA, Caldas C (2005). Molecular Classification and Molecular Forecasting of Breast Cancer: Ready for Clinical Application?. JCO.

[4] Mayer IA, Abramson VG, Lehmann BD, Pietenpol JA (2014). New Strategies for Triple-Negative Breast Cancer—Deciphering the Heterogeneity. Clin Cancer Res.

[5] Morris GJ (2007). Differences in breast carcinoma characteristics in newly diagnosed African-American and Caucasian patients: a single-institution compilation compared with the National Cancer Institute’s Surveillance, Epidemiology, and End Results database. Cancer.

[6] Won K, Spruck C (2020). Triple-negative breast cancer therapy: Current and future perspectives (Review). Int J Oncol.

[7] Dent R, Trudeau M, Pritchard KI (2007). Triple-Negative Breast Cancer: Clinical Features and Patterns of Recurrence. Clin Cancer Res.

[8] Arvold ND, Taghian AG, Niemierko A (2011). Age, Breast Cancer Subtype Approximation, and Local Recurrence After Breast-Conserving Therapy. JCO.

[9] Yin L, Duan J-J, Bian X-W, Yu S (2020). Triple-negative breast cancer molecular subtyping and treatment progress. Breast Cancer Res.

[10] Derakhshan F, Reis-Filho JS (2022). Pathogenesis of Triple-Negative Breast Cancer. Annu Rev Pathol Mech Dis.

[11] Burstein HJ, Curigliano G, Thürlimann B (2021). Customizing local and systemic therapies for women with early breast cancer: the St Gallen International Consensus Guidelines for treatment of early breast cancer. Annals of Oncology..

[12] Curigliano G, Burstein HJ, Winer EP (2017). De-escalating and escalating treatments for early-stage breast cancer: the St gallen international expert consensus conference on the primary therapy of early breast cancer. Annals of Oncology..

[13] Bevers TB, Helvie M, Bonaccio E (2018). Breast Cancer Screening and Diagnosis, Version 3.2018, NCCN Clinical Practice Guidelines in Oncology. J Natl Compr Canc Netw..

[14] Bianchini G, De Angelis C, Licata L, Gianni L (2022). Treatment landscape of triple-negative breast cancer — expanded options, evolving needs. Nat Rev Clin Oncol.

[15] Howard FM, Olopade OI (2021). Epidemiology of Triple-Negative Breast Cancer: A Review. Cancer J.

[16] Loibl S, O’Shaughnessy J, Untch M (2018). Addition of the PARP inhibitor veliparib plus carboplatin or carboplatin alone to standard neoadjuvant chemotherapy in triple-negative breast cancer (BrighTNess): a randomised, phase 3 trial. Lancet Oncol.

[17] Zhang L, Wu Z, Li J (2022). Neoadjuvant docetaxel plus carboplatin vs epirubicin plus cyclophosphamide followed by docetaxel in triple-negative, early-stage breast cancer ( NeoCART ): Results from a multicenter, randomized controlled, open-label phase II trial. Intl Journal of Cancer.

[18] Shepherd JH, Ballman K, Polley MYC (2022). Long term outcomes and genomic correlates of response and survival after neoadjuvant chemotherapy with or without carboplatin and bevacizumab in triple negative breast cancer. J Clin Oncol..

[19] Hahnen E, Lederer B, Hauke J (2017). Germline Mutation Status, Pathological Complete Response, and Disease-Free Survival in Triple-Negative Breast Cancer: Secondary Analysis of the GeparSixto Randomized Clinical Trial. JAMA Oncol.

[20] Schmid P, Cortes J, Pusztai L (2020). Pembrolizumab for Early Triple-Negative Breast Cancer. N Engl J Med.

[21] Cortes J, Rugo HS, Cescon DW (2022). Pembrolizumab plus Chemotherapy in Advanced Triple-Negative Breast Cancer. N Engl J Med.

[22] Gong Y, Ji P, Yang Y-S (2021). Metabolic-Pathway-Based Subtyping of Triple-Negative Breast Cancer Reveals Potential Therapeutic Targets. Cell Metab.

[23] Zhu Y, Zhu X, Tang C, Guan X, Zhang W (2021). Progress and challenges of immunotherapy in triple-negative breast cancer. Biochimica et Biophysica Acta (BBA) - Reviews on Cancer.

[24] Farkona S, Diamandis EP, Blasutig IM (2016). Cancer immunotherapy: the beginning of the end of cancer?. BMC Med.

[25] Nanda R, Liu MC, Yau C (2020). Effect of Pembrolizumab Plus Neoadjuvant Chemotherapy on Pathologic Complete Response in Women With Early-Stage Breast Cancer: An Analysis of the Ongoing Phase 2 Adaptively Randomized I-SPY2 Trial. JAMA Oncol.

[26] LeVasseur N, Sun J, Gondara L (2020). Impact of pathologic complete response on survival after neoadjuvant chemotherapy in early-stage breast cancer: a population-based analysis. J Cancer Res Clin Oncol.

[27] Nanda R, Chow LQM, Dees EC (2016). Pembrolizumab in Patients With Advanced Triple-Negative Breast Cancer: Phase Ib KEYNOTE-012 Study. JCO.

[28] Cortazar P, Zhang L, Untch M (2014). Pathological complete response and long-term clinical benefit in breast cancer: the CTNeoBC pooled analysis. The Lancet.

[29] Roodhart J, Langenberg M, Witteveen E, Voest E (2008). The Molecular Basis of Class Side Effects Due to Treatment with Inhibitors of the VEGF/VEGFR Pathway. CCP.

[30] Syrigos KN, Karapanagiotou E, Boura P, Manegold C, Harrington K (2011). Bevacizumab-Induced Hypertension: Pathogenesis and Management. BioDrugs.

[31] Liu J, Liu Q, Li Y, et al. Efficacy and safety of camrelizumab combined with apatinib in advanced triple-negative breast cancer: an open-label phase II trial[J]. J Immunother Cancer. 2020, 8(1). 10.1136/jitc-2020-000696 PMC7252975 32448804

[32] Yi M, Jiao D, Qin S, Chu Q, Wu K, Li A (2019). Synergistic effect of immune checkpoint blockade and anti-angiogenesis in cancer treatment. Mol Cancer.

[33] Liberati A, Altman DG, Tetzlaff J (2009). The PRISMA Statement for Reporting Systematic Reviews and Meta-Analyses of Studies That Evaluate Health Care Interventions: Explanation and Elaboration. PLoS Med.

[34] Iwase M, Ando M, Aogi K (2020). Long-term survival analysis of addition of carboplatin to neoadjuvant chemotherapy in HER2-negative breast cancer. Breast Cancer Res Treat.

[35] Chen X, Ye G, Zhang C (2013). Superior outcome after neoadjuvant chemotherapy with docetaxel, anthracycline, and cyclophosphamide versus docetaxel plus cyclophosphamide: results from the NATT trial in triple negative or HER2 positive breast cancer. Breast Cancer Res Treat.

[36] Untch M, Von Minckwitz G, Konecny GE (2011). PREPARE trial: a randomized phase III trial comparing preoperative, dose-dense, dose-intensified chemotherapy with epirubicin, paclitaxel, and CMF versus a standard-dosed epirubicin–cyclophosphamide followed by paclitaxel with or without darbepoetin alfa in primary breast cancer—outcome on prognosis. Ann Oncol.

[37] Sharma P, Kimler BF, O’Dea A (2021). Randomized Phase II Trial of Anthracycline-free and Anthracycline-containing Neoadjuvant Carboplatin Chemotherapy Regimens in Stage I-III Triple-negative Breast Cancer (NeoSTOP). Clin Cancer Res.

[38] Zhang L, Wu Z, Li J (2021). Neoadjuvant Docetaxel plus Carboplatin Versus Epirubicin plus Cyclophosphamide Followed by Docetaxel in Triple-negative, Early-stage Breast Cancer (NeoCART): Results from a Multicenter, Randomized Controlled. Open-label Phase II Trial In Review.

[39] Schneeweiss A, Michel LL, Möbus V (2022). Survival analysis of the randomised phase III GeparOcto trial comparing neoadjuvant chemotherapy of intense dose-dense epirubicin, paclitaxel, cyclophosphamide versus weekly paclitaxel, liposomal doxorubicin (plus carboplatin in triple-negative breast cancer) for patients with high-risk early breast cancer. Eur J Cancer.

[40] Gluz O, Nitz U, Kolberg-Liedtke C (2022). De-escalated Neoadjuvant Chemotherapy in Early Triple-Negative Breast Cancer (TNBC): Impact of Molecular Markers and Final Survival Analysis of the WSG-ADAPT-TN Trial. Clin Cancer Res.

[41] Zhang P, Yin Y, Mo H (2016). Better pathologic complete response and relapse-free survival after carboplatin plus paclitaxel compared with epirubicin plus paclitaxel as neoadjuvant chemotherapy for locally advanced triple-negative breast cancer: a randomized phase 2 trial. Oncotarget.

[42] Yan W, Wu X, Wang S (2022). Lobaplatin-based neoadjuvant chemotherapy for triple-negative breast cancer: a 5-year follow-up of a randomized, open-label, phase II trial. Ther Adv Med Oncol.

[43] Schmid P, Salgado R, Park YH (2020). Pembrolizumab plus chemotherapy as neoadjuvant treatment of high-risk, early-stage triple-negative breast cancer: results from the phase 1b open-label, multicohort KEYNOTE-173 study. Ann Oncol.

[44] Schmid P, Cortes J, Dent R (2022). Event-free Survival with Pembrolizumab in Early Triple-Negative Breast Cancer. N Engl J Med.

[45] Loibl S, Schneeweiss A, Huober J (2022). Neoadjuvant durvalumab improves survival in early triple-negative breast cancer independent of pathological complete response. Ann Oncol.

[46] Bear HD, Tang G, Rastogi P (2015). Neoadjuvant plus adjuvant bevacizumab in early breast cancer (NSABP B-40 [NRG Oncology]): secondary outcomes of a phase 3, randomised controlled trial. Lancet Oncol.

[47] Von Minckwitz G, Loibl S, Untch M (2014). Survival after neoadjuvant chemotherapy with or without bevacizumab or everolimus for HER2-negative primary breast cancer (GBG 44–GeparQuinto). Ann Oncol.

[48] Nahleh ZA, Barlow WE, Hayes DF (2016). SWOG S0800 (NCI CDR0000636131): addition of bevacizumab to neoadjuvant nab-paclitaxel with dose-dense doxorubicin and cyclophosphamide improves pathologic complete response (pCR) rates in inflammatory or locally advanced breast cancer. Breast Cancer Res Treat.

[49] Earl HM, Hiller L, Dunn JA (2017). Disease-free and overall survival at 3.5 years for neoadjuvant bevacizumab added to docetaxel followed by fluorouracil, epirubicin and cyclophosphamide, for women with HER2 negative early breast cancer: ARTemis Trial. Annals of Oncology..

[50] Poggio F, Bruzzone M, Ceppi M (2018). Platinum-based neoadjuvant chemotherapy in triple-negative breast cancer: a systematic review and meta-analysis. Ann Oncol.

[51] Li Y, Yang D, Chen P (2019). Efficacy and safety of neoadjuvant chemotherapy regimens for triple-negative breast cancer: a network meta-analysis. Aging.

[52] Yin J, Zhu C, Wang G, Gu J (2022). Treatment for Triple-Negative Breast Cancer: An Umbrella Review of Meta-Analyses. IJGM.

[53] Li J, Shen G, Wang M (2022). Comparative efficacy and safety of first-line neoadjuvant treatments in triple-negative breast cancer: systematic review and network meta-analysis. Clin Exp Med.

[54] Alnimer Y, Hindi Z, Katato K (2018). The Effect of Perioperative Bevacizumab on Disease-Free and Overall Survival in Locally Advanced HER-2 Negative Breast Cancer: A Meta-Analysis. Breast Cancer(Auckl).

[55] Ma X, Wang X, Huang J (2016). Bevacizumab Addition in Neoadjuvant Treatment Increases the Pathological Complete Response Rates in Patients with HER-2 Negative Breast Cancer Especially Triple Negative Breast Cancer: A Meta-Analysis. PLoS ONE.

[56] Nahleh Z, Botrus G, Dwivedi A, Jennings M, Nagy S, Tfayli A. Bevacizumab in the neoadjuvant treatment of human epidermal growth factor receptor 2-negative breast cancer: A meta-analysis of randomized controlled trials. mol clin onc 2019. Published online Jan 2. 10.3892/mco.2019.1796. 10.3892/mco.2019.1796 PMC6388502 30847174

[57] Li Z-Y, Zhang Z, Cao X-Z, Feng Y, Ren S-S (2020). Platinum-based neoadjuvant chemotherapy for triple-negative breast cancer: a systematic review and meta-analysis. J Int Med Res.

[58] Petrelli F, Coinu A, Borgonovo K (2014). The value of platinum agents as neoadjuvant chemotherapy in triple-negative breast cancers: a systematic review and meta-analysis. Breast Cancer Res Treat.

[59] Sternschuss M, Yerushalmi R, Saleh RR, Amir E, Goldvaser H (2021). Efficacy and safety of neoadjuvant immune checkpoint inhibitors in early-stage triple-negative breast cancer: a systematic review and meta-analysis. J Cancer Res Clin Oncol.

